# Wrist Salvage in a Transscaphoid Transcapitate Perilunate Fracture-Dislocation in the Setting of Previous Scaphoid Nonunion and Scaphoid Nonunion Advanced Collapse (SNAC)

**Published:** 2020-05-11

**Authors:** Sean J. Wallace, Ethan Song, Nathan F. Miller, Lawrence E. Weiss

**Affiliations:** ^a^Division of Plastic & Reconstructive Surgery, Department of Surgery, Lehigh Valley Health Network, Allentown, Pa; ^b^Morsani College of Medicine, University of South Florida, Tampa; ^c^Division of Orthopedic Hand Surgery, OAA Orthopedic Specialists, Allentown, Pa

**Keywords:** perilunate dislocation, Mayfield's classification, scaphoid nonunion, scaphoid nonunion advanced collapse, wrist salvage

## DESCRIPTION

A 59-year-old man fell onto his outstretched hands. He was diagnosed with a left-sided transscaphoid transcapitate perilunate fracture-dislocation in the setting of previous scaphoid open reduction internal fixation (ORIF), resulting in a nonunion and scaphoid nonunion advanced collapse (SNAC). Wrist salvage in the form of primary partial wrist fusion was performed and resulted in excellent clinical and functional outcomes.

## QUESTIONS

1. What are perilunate dislocations and/or perilunate fracture-dislocations?
2. What physical and radiologic examination findings are typically encountered with perilunate dislocations and fracture-dislocations?
3. How are perilunate dislocations and fracture-dislocations managed?
4. When a perilunate fracture-dislocation presents in the setting of previous scaphoid nonunion and SNAC wrist, what is the best surgical option to give the best functional outcome?

## DISCUSSION

Perilunate dislocations and fracture-dislocations are debilitating wrist injuries resulting from high-energy trauma. Dislocations can be purely ligamentous or involve a combination of bony and ligamentous structures. When involving only ligaments surrounding the lunate, these dislocations are referred to as lesser arc injuries. When the dislocation is associated with a radial styloid or carpal bone fractures, they are referred to as greater arc injuries.^1^ A stepwise injury progression has been previously described by Mayfield et al.^2^ Ligamentous injuries commonly progress from radial to ulnar, disrupting the scapholunate ligament first. As the force transmits, ulnar, capitolunate, and lunotriquetral articulations are disrupted sequentially, which leads to failure of the dorsal radiocarpal ligament and dislocation of the lunate from the lunate fossa. When greater arc injuries occur, the force transmits through the radial styloid and carpus. These injuries commonly result in transscaphoid perilunate fracture-dislocations, but they can also involve the radial styloid or other carpal bones.^3^ Despite proper reconstruction, outcomes for perilunate dislocations and perilunate fracture-dislocations are notoriously poor. Patients often develop decreased grip strength and range of motion. Patients may go on to develop traumatic arthritis of the wrist requiring wrist salvage procedures such as 4-corner fusion or proximal row carpectomy.^4^

**Figure 1:**
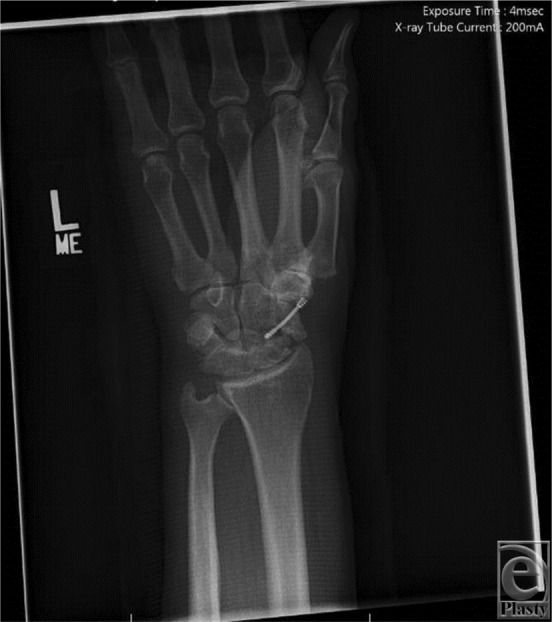
Preoperative posteroanterior radiograph of transscaphoid transcapitate perilunate fracture-dislocation demonstrating previous scaphoid open reduction internal fixation resulting in nonunion and scaphoid nonunion advanced collapse wrist. Also demonstrated are loss of carpal height and alternation of Gilula's lines.

**Figure 2:**
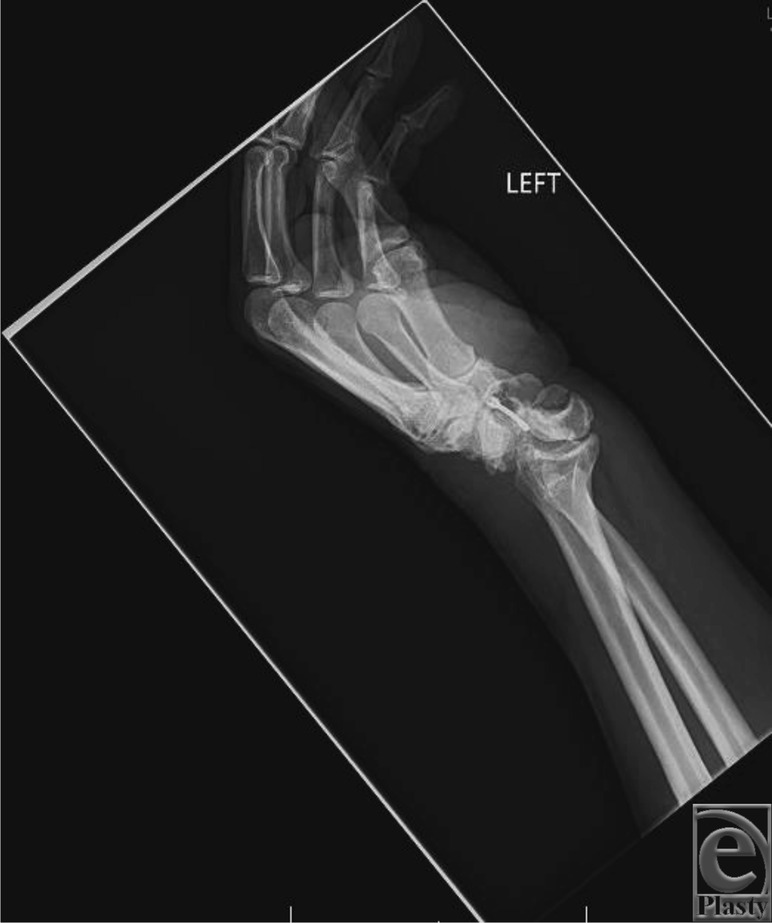
Preoperative lateral radiograph of transscaphoid transcapitate perilunate fracture-dislocation in the setting of scaphoid nonunion and scaphoid nonunion advanced collapse demonstrating posterior displacement of the capitate relative to the lunate.

Physical examination often reveals a grossly swollen wrist with limited range of motion with or without signs of acute median neuropathy. The presence of acute carpal tunnel syndrome is an indication for urgent operative intervention if closed reduction has failed. Posteroanterior radiographic evaluation may demonstrate alteration of Gilula's lines with loss of carpal height (Fig 1). There may also be widening of the scapholunate interval with an associated cortical ring sign of the scaphoid. On a lateral radiograph, the capitate may be displaced posterior relative to the lunate, disrupting the collinear relationship between the radius, lunate, and capitate (Fig 2).

Surgical correction involves acute carpal tunnel decompression, relocation of the carpus, repair of the capsular rent, and stabilization of the carpus with percutaneous pin fixation. Controversy exists whether acute scapholunate ligament reconstruction should be performed.^4^ In the setting of scaphoid fractures associated with the injury, the scaphoid is often stabilized with cannulated screw fixation with or without autologous bone grafting. However, because of the retrograde blood supply of the scaphoid to the proximal pole, scaphoid fractures may lead to nonunion or malunion. Unrepaired scaphoid fractures may develop SNAC and can progress to pan-carpal arthritis. Wrist salvage procedures are indicated when the arthritis has advanced to a degree of which the patient cannot tolerate the pain.^4^^,^^5^

**Figure 3:**
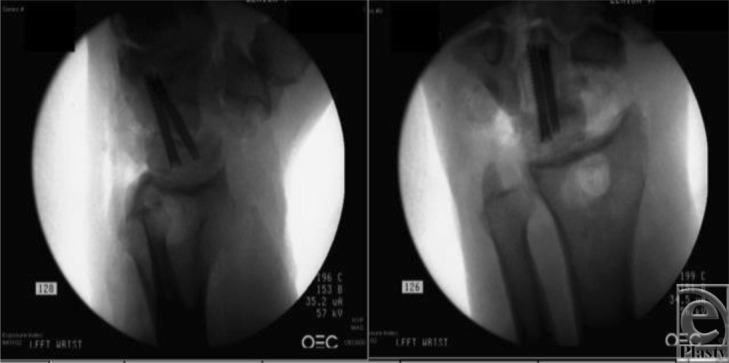
Intraoperative fluoroscopic view after reduction and primary partial midcarpal wrist arthrodesis.

**Figure 4:**
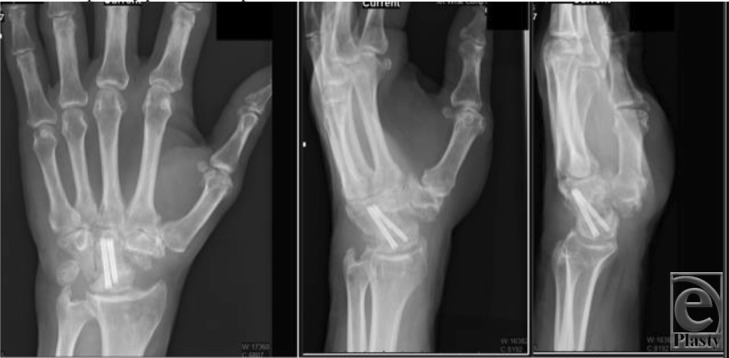
Postoperative posteroanterior, lateral, and oblique radiographs at 4 months revealing consolidation of primary partial midcarpal wrist arthrodesis.

We present a case of a patient who suffered a transscaphoid transcapitate perilunate fracture-dislocation in the setting of a prior scaphoid nonunion and SNAC wrist. Radiographs revealed a greater arc injury with transmission of force through the previous scaphoid nonunion site, resulting in displacement of the cannulated screw from the proximal pole of the scaphoid (Figs 1 and 2). Scaphoid nonunion was suspected on radiographs and computed tomographic scan and was confirmed with intraoperative evaluation that revealed sclerotic bone with avascular changes within the proximal pole and a displaced previously placed surgical screw transfixing the nonunion site. Visual intraoperative confirmation of the SNAC was evidenced by degenerative changes at the radioscaphoid joint and radial styloid, as well as significant midcarpal joint arthrosis (Fig 1). In this setting, we theorized that a primary wrist salvage procedure would provide the best outcome. A standard ORIF would leave the midcarpal arthrosis unresolved. Given the presence of a proximal capitate fracture and concomitant midcarpal arthrosis, a midcarpal arthrodesis was favored over proximal row carpectomy. To eliminate the amount of fusion that needed to occur, we excised the proximal pole of the capitate. The shortened capitate created certain challenges in terms of considering the standard scaphoidectomy and 4-corner fusion. To adjust for the decreased height of the capitate in relation to the triquetral-hamate articulation, the triquetrum was excised. This afforded the opportunity to fuse the shortened capitate to the lunate using a headless screw along with distal radius bone graft (Fig 3). Ultimately, that patient developed 40° of flexion and extension of both wrists, full digital flexion, and consolidation of fusion (Fig 4) and was able to return to work, performing all of his previous activities.
